# Supramolecular self-assembled nanomedicine targeting fatty acid metabolism to potentiate ferroptosis-enhanced photoimmunotherapy in triple-negative breast cancer

**DOI:** 10.1016/j.mtbio.2026.103748

**Published:** 2026-10-12

**Authors:** Fei Li, Junyong Wu, Xinyan Hao, Wenjie Xu, Jiemin Wang, Jiwen Zhang, Xingyu Wei, Niwen Feng, Fangyuan Liu, Dehua Liao

**Affiliations:** a Department of Pharmacy, Hunan Cancer Hospital, the Affiliated Cancer Hospital of Xiangya School of Medicine, Central South University, Changsha, 410013, China; b School of Pharmacy, University of South China, Hengyang, 421099, China; c Department of Pharmacy, The Second Xiangya Hospital, Central South University, Changsha, 410011, China; d Comprehensive Health Research Centre (CHRC), NOVA Medical School, Faculdade de Ciências Médicas, NMS|FCM, Universidade NOVA de Lisboa, Lisboa, Portugal

**Keywords:** Self-assembled nanomedicine, Photodynamic immunotherapy, Ferroptosis, Lipid metabolism, Triple-negative breast cancer, Tumor microenvironment

## Abstract

Triple-negative breast cancer (TNBC) presents a serious therapeutic challenge, largely owing to its metabolic adaptability and the immunosuppressive nature of its tumor microenvironment (TME). Photodynamic therapy (PDT) can cause oxidative injury and induce immunogenic cell death (ICD); however, its antitumor activity is often limited by inadequate reactive oxygen species (ROS) accumulation and weak immune activation. To address these barriers, a supramolecular self-assembled nanoplatform, termed OC@cRGD, was constructed through the co-assembly of the fatty acid synthase (FASN) inhibitor orlistat and the photosensitizer chlorin e6 (Ce6), followed by cyclic RGD (cRGD) modification to support integrin-mediated tumor targeting. This self-assembled design allows high drug loading and links metabolic interference with redox stress in a single nanoscale formulation. At the mechanistic level, orlistat disturbs lipid homeostasis and reduces the saturated fatty acid–based protection that helps TNBC cells resist membrane peroxidation. In parallel, light-activated PDT delivers a spatially restricted oxidative burst. After laser irradiation, these two processes act together to drive extensive iron-dependent lipid peroxidation, along with reduced GPX4 and FASN expression, thereby increasing cellular susceptibility to ferroptosis. This ferroptosis-reinforced PDT further induces pronounced ICD. In 4T1 tumor-bearing mice, OC@cRGD combined with laser irradiation inhibited tumor growth and reshaped the immune microenvironment, as reflected by dendritic cell maturation, M1-like macrophage polarization, and increased CD8^+^ T-cell infiltration. Overall, this study combines metabolic intervention with photoimmunotherapy and offers a metabolism-guided strategy for the development of self-assembled nanotherapeutics.

## Graphical abstract

**Figure:**
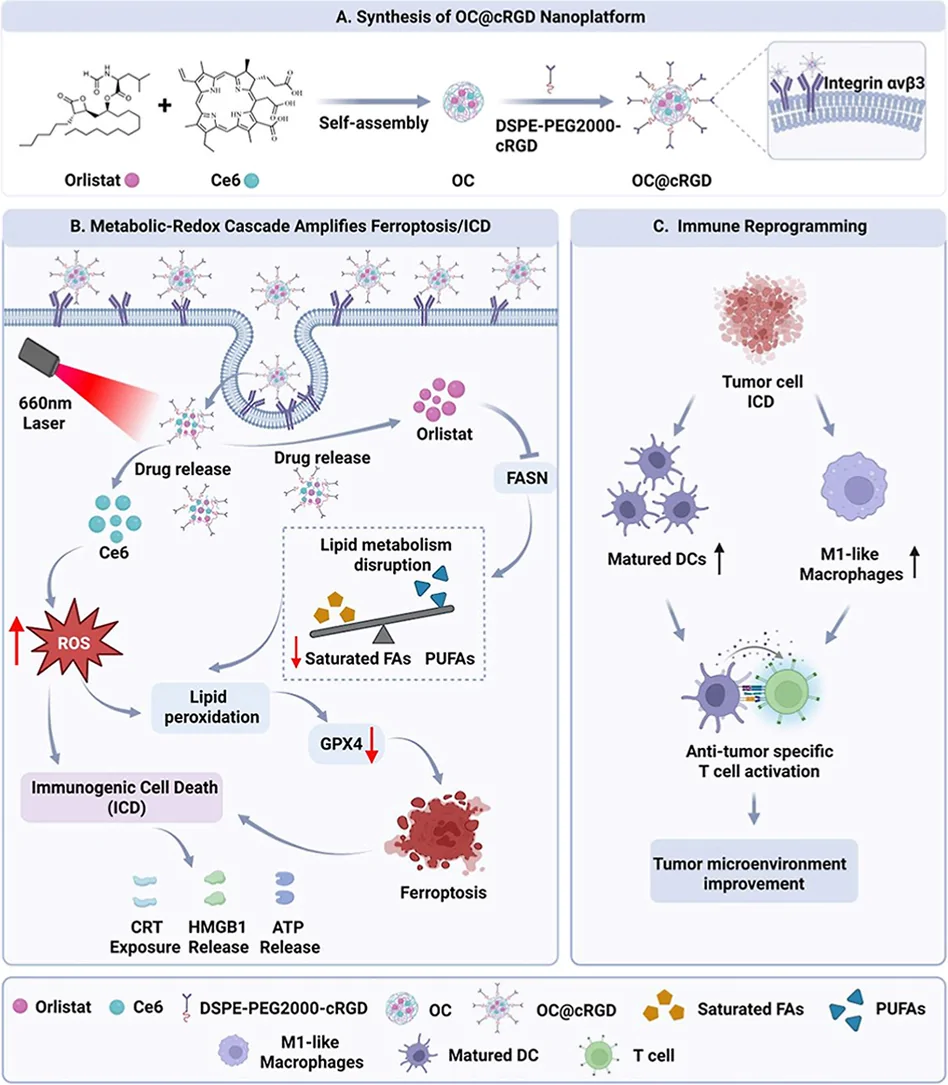

## 1 Introduction

Triple-negative breast cancer (TNBC) persists as the most aggressive breast cancer subtype, posing significant challenges for clinical treatment [1]. TNBC lacks estrogen receptor and progesterone receptor expression and does not show HER2 amplification; consequently, many molecularly targeted therapies have little therapeutic value, and chemotherapy still serves as the principal treatment option [2], [3], [4]. In recent years, immune-based therapy has broadened the therapeutic repertoire, yet only a limited subset of patients derives sustained benefit. This restricted response is closely associated with the strong metabolic adaptability of TNBC and its immunosuppressive tumor microenvironment [5], [6], [7]. Therefore, developing therapeutic strategies that simultaneously induce direct tumor cell death and elicit systemic antitumor immunity is highly desirable (see Scheme 1).

**Scheme 1:**
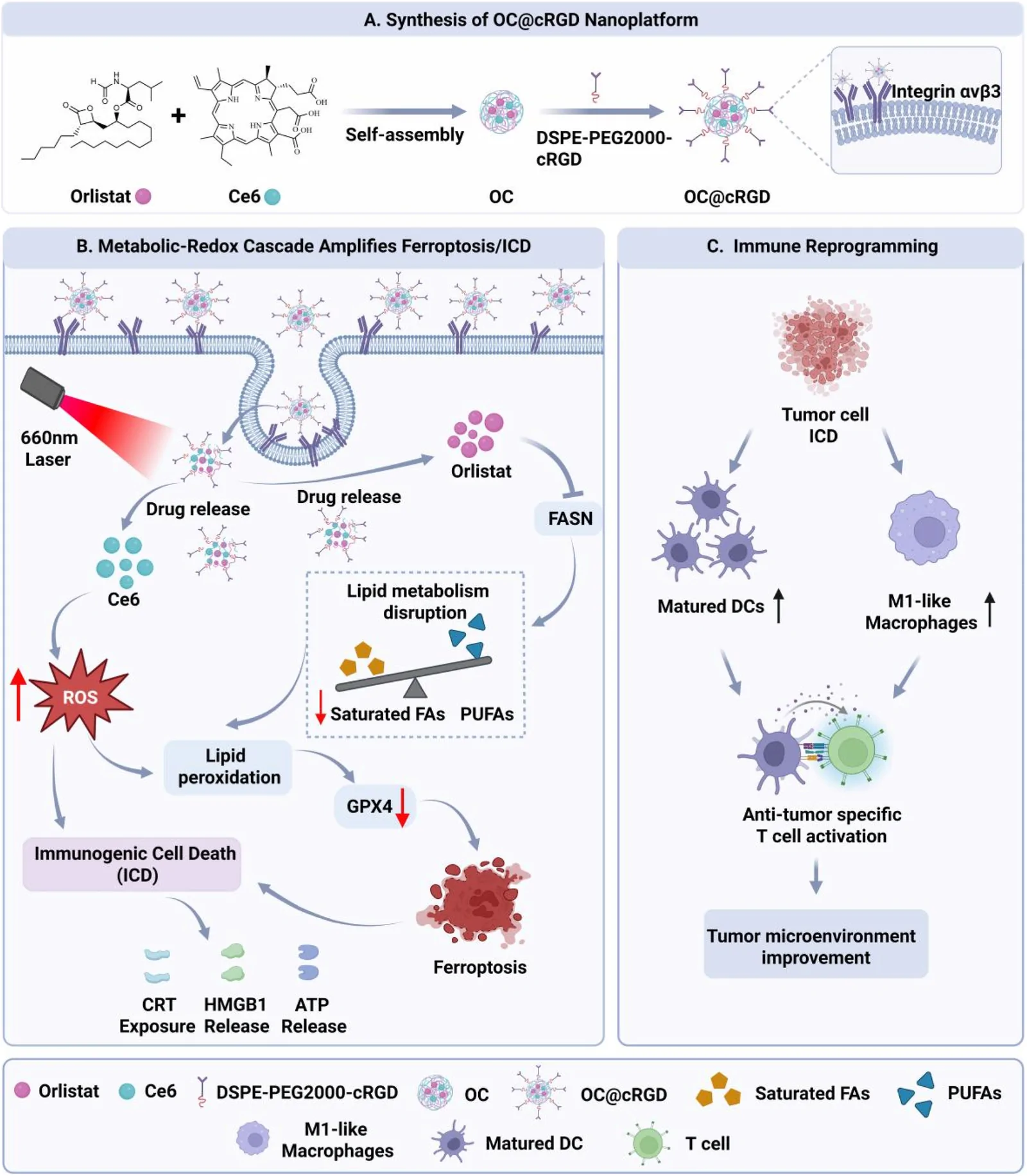
Schematic illustration of the OC@cRGD nanoplatform for enhanced tumor therapy. (A) Orlistat and Ce6 self-assembled with DSPE-PEG2000-cRGD to form an integrin αvβ3 targeted self-assembled nanoplatform. (B) Upon 660 nm laser irradiation, Ce6 generates ROS to trigger lipid peroxidation, while orlistat inhibits FASN to disrupt lipid metabolism. This metabolic-redox cascade amplifies ferroptosis and triggers ICD. (C) ICD amplifies *in vivo* immune activation, thereby promoting DC maturation, M1 macrophage polarization, and T cell-mediated anti-tumor immunity.

Photodynamic therapy (PDT) offers a non-invasive and spatially controllable treatment modality with the ability to induce immunogenic cell death (ICD) [8], [9]. After light exposure, photosensitizers produce reactive oxygen species (ROS), which damage organelles and biological macromolecules and eventually lead to cell death [10], [11], [12]. This oxidative injury also promotes the release or exposure of danger-associated signals, including calreticulin (CRT), ATP, and HMGB1. These signals facilitate dendritic cell maturation and subsequent T-cell activation, potentially converting immunologically “cold” tumors into “hot” ones [13], [14]. However, the therapeutic efficacy of PDT is often limited by intracellular antioxidant systems. In particular, the glutathione network rapidly neutralizes ROS, thereby restricting the extent of oxidative injury [10], [15].

Ferroptosis, an iron-dependent form of regulated cell death, results from excessive lipid peroxide accumulation and proceeds independently of classical apoptosis [16], [17], [18], [19], [20]. Because the membrane lipid peroxidation characteristic of ferroptosis shares the oxidative damage pathway initiated by PDT-generated ROS, combining these two modalities provides a rational synergistic approach. Activation of ferroptosis can increase PDT-induced cytotoxicity and intensify ICD-associated danger signaling [21], [22], [23]. Mechanistically, ferroptosis depends on the inactivation of the GPX4/glutathione (GSH) system. This inactivation impairs the clearance of lipid peroxides, facilitating the propagation of PDT-induced oxidative damage. However, cellular susceptibility to ferroptosis depends on both oxidative stress levels and membrane lipid composition, the latter being regulated by fatty acid metabolism [24], [25], [26]. Fatty acid synthase (FASN), which is frequently upregulated in TNBC, is the rate-limiting enzyme governing *de novo* lipogenesis [27], [28]. The saturated fatty acids synthesized by FASN lack unsaturated double bonds, making them structurally resistant to lipid peroxidation. This lipid profile may give TNBC cells an intrinsic resistance to ferroptosis and reduce the efficacy of therapies that combine PDT with ferroptotic induction [29], [30], [31]. Our analysis of transcriptomic data from the GSE58812 TNBC cohort supports this hypothesis: FASN expression positively correlated with the ferroptosis suppressor GPX4 and negatively correlated with the ferroptosis driver ACSL4 (Fig. S1). These correlations suggest that FASN mediated lipogenesis maintains a lipid environment unfavorable for ferroptosis. Therefore, pharmacological inhibition of FASN may lower the threshold for lipid peroxidation and sensitize TNBC cells to PDT.

Orlistat, an FDA approved FASN inhibitor, disrupts lipid metabolic activity in tumor cells [32]. We hypothesized that co-delivering orlistat with a photosensitizer could integrate metabolic regulation with light-triggered oxidative stress. Specifically, FASN inhibition would shift the cellular fatty acid pool by decreasing peroxidation-resistant saturated lipids, whereas PDT would supply ROS to promote ferroptotic injury. Together, these effects are expected to strengthen direct tumor cytotoxicity and increase subsequent immune activation. A technical challenge for this combination therapy is the co-delivery of molecules with distinct physicochemical properties. To address this issue, self-assembled drug delivery systems provide an attractive formulation strategy. In general, carrier-free self-assembled nanomedicines refer to nanosystems constructed entirely or predominantly from active therapeutic molecules themselves, without the use of conventional inert carrier matrices for drug encapsulation. Such systems can provide high drug loading, simplify preparation, and reduce carrier-associated toxicity [33], [34], [35]. Their self-assembly is usually driven by noncovalent interactions, including π–π stacking, hydrophobic interactions, hydrogen bonding, and other intermolecular forces [36]. However, when auxiliary components are introduced for colloidal stabilization, surface engineering, or active targeting, the resulting formulations should be more accurately described as drug-based self-assembled nanosystems rather than strictly carrier-free nanomedicines. In the current design, chlorin e6 (Ce6) possesses a conjugated porphyrin macrocycle that enables intermolecular π–π stacking, whereas orlistat carries hydrophobic alkyl chains that favor hydrophobic association. In addition, hydrogen bonding between the functional groups of Ce6 and orlistat further contributes to the stabilization of the co-assembled structure. Driven by these cooperative noncovalent interactions, the two therapeutic molecules spontaneously co-assemble into stable OC nanoassemblies. To further improve tumor-targeted delivery, DSPE-PEG2000-cRGD was introduced as a surface-functionalizing component to confer integrin αvβ3-targeting ability, as integrin αvβ3 is frequently overexpressed in TNBC cells [37], [38]. Therefore, the final formulation, termed OC@cRGD, is defined in this study as a cRGD-decorated drug-based self-assembled nanoplatform rather than a strictly carrier-free self-assembled nanomedicine. This nanoplatform was designed to improve tumor accumulation through active targeting. After cellular internalization, the nanoplatform aims to suppress FASN mediated lipid metabolism, thereby sensitizing cells to ferroptosis. Subsequent 660 nm laser irradiation provides the oxidative stress necessary to trigger ferroptotic cell death and initiate antitumor immune responses. In this study, we evaluated the capacity of OC@cRGD to remodel lipid metabolism, enhance Ce6-mediated photodynamic toxicity, and induce ICD in TNBC cells. Furthermore, we assessed its effect on dendritic cell maturation and CD8^+^ T-cell infiltration. By integrating metabolic intervention and photoimmunotherapy within a self-assembled nanosystem, this work provides a potential metabolism-guided strategy for TNBC treatment.

## 2 Materials and methods

### 2.1 Materials

Orlistat and Ce6 (Aladdin Biochemical Technology, Shanghai); DSPE-PEG2000-cRGD (RuixiBio, Xi'an); Urea, NaCl, and dimethyl sulfoxide (DMSO) (Sinopharm Chemical Reagent Co., Ltd., Shanghai); Vitamin E polyethylene glycol succinate (TPGS) (Shanghai Macklin Biochemical Co., Ltd.); D-α-tocopheryl succinate (α-TOS) (Yuanye Biotechnology Co., Ltd., Shanghai); Ferrostatin-1 (Fer-1) (MCE, Shanghai); DAPI Staining Reagent (Servicebio, Wuhan); DiR (Yeasen Biotechnology, Shanghai); Cell Counting Kit-8 (CCK-8) (EnoGene, Nanjing); Calcein-AM/PI kit, ROS detection kit (Yeasen Biotechnology Co., Ltd.); FerroOrange Staining Kit (MKBio, Shanghai); β-actin and GPX4 antibodies (HUABIO, Hangzhou); FASN and CD61 antibodies (Abiowell, Changsha); fetal bovine serum (Vazyme Biotech Co., Ltd., Nanjing); Dulbecco's modified Eagle's medium (DMEM) and RPMI-1640 medium (Wuhan Procell Biotechnology Co., Ltd.).

### 2.2 Preparation of OC and OC@cRGD

Ce6 (2 mg) and orlistat (2 mg) were dissolved together in 200 μL DMSO containing α-TOS (0.4 mg) and TPGS (0.1 mg). TPGS, an FDA-approved pharmaceutical excipient, was used to reduce interfacial tension and enhance nanoparticle stability via steric stabilization due to its amphiphilic structure [39], [40]. α-TOS was included to further stabilize the nanoparticles through hydrophobic interactions. The whole procedure was carried out under ambient conditions and protected from light. After 5 min of sonication, the mixture formed a clear and homogeneous organic solution. This solution was added slowly into 4.8 mL ultrapure water under continuous magnetic stirring at approximately 1000 rpm. One drop was added every 5 s. During this process, self-assembled nanoparticles formed spontaneously and were named OC. For surface modification of the freshly prepared OC dispersion, 1 mg of DSPE-PEG2000-cRGD was gradually introduced. The resulting mixture was then stirred in the dark at room temperature for an additional 15 min. Subsequently, the suspension was placed into a dialysis membrane bag (MWCO 10 kDa) and dialyzed against deionized water for 24 h, thereby eliminating residual DMSO and any unassembled free components.

### 2.3 Characterization of OC and OC@cRGD

The structural features of OC and OC@cRGD were characterized by transmission electron microscopy (TEM), its hydrodynamic particle size and surface charge were determined using a Zetasizer Nano ZS90 dynamic light scattering instrument (Malvern, UK). To assess dispersion stability, OC and OC@cRGD were incubated in PBS, 10% FBS, 5% glucose, NaCl solution (0.4M), urea solution (0.4M), and SDS solution (0.4%, w/v). Changes in hydrodynamic diameter and absorbance were then recorded. The amount of surface-modified cRGD was quantified using a bicinchoninic acid (BCA) assay according to a previously reported method [41]. Briefly, 20 μL of each cRGD standard solution or unknown sample was added to a 96-well plate and mixed with 200 μL of BCA working reagent. After incubation at 37°C for 4 h, the absorbance was measured at 562 nm using a microplate reader. A standard curve was constructed using cRGD solutions at concentrations ranging from 0 to 150 μg/mL. To remove unbound cRGD, dialyzed OC and OC@cRGD dispersions were further processed by ultrafiltration. The retained nanoparticles were then re-dispersed in ultrapure water and subjected to BCA analysis. High-performance liquid chromatography (HPLC) was used to quantitatively analyze Ce6 and orlistat based on the corresponding external standard calibration curves. Drug loading capacity and encapsulation efficiency were derived from the measured drug content using the stated equations.

### 2.4 Cell culture and animals

4T1 cells were maintained in DMEM supplemented with 10% FBS and 1% penicillin–streptomycin, while MCF-10A cells were cultured in a specialized medium, at 37°C in a humidified atmosphere containing 5% CO_2_. Female BALB/c mice were obtained from SJA Laboratory Animal Co., Ltd. (Changsha, China), and the study was approved by the Institutional Animal Care and Use Committee (IACUC) of Xiangya Second Hospital, Central South University.

### 2.5 *In vitro* cellular uptake

4T1 cells were cultured for 24 h and then incubated with free Ce6, OC, or OC@cRGD (equal Ce6 concentration) for 6 h. For CLSM (Stellaris 5, Leica, Germany), cells were washed, fixed with 4% paraformaldehyde, and mounted. For flow cytometry, cells were collected, washed, centrifuged, resuspended, and analyzed for Ce6 fluorescence intensity. Both methods relied on Ce6 autofluorescence to assess cellular uptake.

### 2.6 Cell viability assay

4T1 cells in 96-well plates were cultured for 24 h, then treated with free orlistat, free Ce6, OC, or OC@cRGD for 8 h. Some groups received 660 nm laser (100 mW/cm^2^, 2 min), while the rest remained unirradiated. After 16 h, CCK-8 assay was used to measure cell toxicity.

### 2.7 Live-dead cell staining

To examine the effects of different treatments on the survival rate of 4T1 cells, fluorescence detection was performed using the calcein AM/PI dual-staining method. 4T1 cells were seeded in 24-well plates and cultured for 24 h; the cells were then treated for 12 h respectively with free orlistat, free Ce6, OC, or OC@cRGD at the same concentrations. For groups receiving irradiation, a 660 nm laser (100 mW/cm^2^, 2 min) was applied; non-irradiated groups were treated identically but without light exposure. After an additional 2 h, the cells were washed with PBS, stained with Calcein-AM/PI for 20 min according to the manufacturer's instructions, and visualized using a fluorescence microscope.

### 2.8 Colony-forming assay

4T1 cells were plated in 6-well plates (1000/well) and cultured for 24 h, then treated with the indicated agents for 12 h, with laser applied to irradiated groups. Medium was replaced with fresh complete medium, and incubation continued until colonies appeared. Colonies were then washed, fixed with 4% paraformaldehyde, stained with crystal violet, and counted to assess long-term proliferation.

### 2.9 Intracellular ROS detection

ROS levels were measured using a DCFH-DA probe. For microscopy, 4T1 cells were treated with free orlistat, free Ce6, OC, or OC@cRGD for 12 h. Only the designated irradiation groups received 660 nm laser exposure at 100 mW/cm^2^ for 2 min; the non-irradiation groups were maintained in the dark. The cells were incubated with DCFH-DA in serum-free medium (30 min, 37°C, in the dark) after an additional period, then washed with PBS and imaged. For flow cytometry, cells in 6-well plates underwent the same treatment and staining, and intracellular fluorescence was analyzed.

### 2.10 Ferroptosis rescue assay

A rescue assay in 96-well plates was used to determine whether ferroptosis mediated OC@cRGD cytotoxicity. After seeding 4T1 cells were cultured in adhesion for 24 h, pretreated with 10 μM ferroptosis inhibitor for 2 h, and then co-incubated with OC@cRGD at different concentrations for 8 h. The cells were then divided into two groups: one group was exposed to 660 nm laser irradiation (100 mW/cm^2^, 2 min), while the other group was kept in the dark. After continuing the culture for 16 h, cell viability was assayed according to the instructions in the CCK-8 assay kit.

### 2.11 Lipid peroxidation assay

Assessment of lipid peroxidation employed the C11-BODIPY 581/591 probe. For confocal imaging, 4T1 cells, after being seeded in confocal dishes and cultured for 24 h, were subjected to a 12 h incubation with free orlistat, free Ce6, OC, or OC@cRGD. Only irradiation-designated samples received 660 nm laser irradiation at 100 mW/cm^2^ for 2 min and further incubation. Staining with the probe was done at 37°C in the dark (manufacturer's protocol). Confocal microscopy detected fluorescence; red-to-green conversion indicated lipid peroxidation. For flow cytometry, cells in 6-well plates were treated, stained, harvested, and analyzed identically.

### 2.12 Intracellular Fe^2+^ detection assay

Cellular ferrous ion levels were monitored with a FerroOrange staining kit. For imaging, 4T1 cells were initially placed in confocal dishes and cultured for 24 h before any treatment, after which free orlistat, free Ce6, OC, or OC@cRGD were added for 12 h. In the designated groups, 660 nm laser exposure was applied at 100 mW/cm^2^ for 2 min, followed by additional incubation. Subsequently, cells from the different treatment groups were collected and stained with FerroOrange in the dark according to the manufacturer's instructions. Fluorescence images were then acquired using a CLSM.

### 2.13 Western blot

GPX4 and FASN protein expression were determined by Western blotting. After seeding 4T1 cells in a 6-well plate and culturing them for 24 h, the cells were treated with the drug. After an additional 24 h, total protein was extracted using RIPA lysis buffer containing PMSF, and the concentration was determined and quantified using the BCA method. Equal volumes of protein samples were loaded onto SDS-PAGE gels; after electrophoretic separation, the proteins were transferred to membranes. The membranes were blocked with 5% skim milk at room temperature for 1 h, followed by overnight incubation at 4°C with primary antibodies against GPX4, FASN, and β-actin. After thorough washing with TBST, the membrane was incubated with secondary antibodies at room temperature for 1 h, washed again with TBST, and the bands were quantified after development. The expression levels of GPX4 and FASN were normalized using β-actin as an internal control.

### 2.14 Analysis of ICD

4T1 cells were seeded onto coverslips in 24-well plates, cultured for 24 h, and then treated with free orlistat, free Ce6, OC, or OC@cRGD for 12 h. A subset received 660 nm laser irradiation at 100 mW/cm^2^ for 2 min and was further incubated for 6 h. For HMGB1 staining, cells were fixed, permeabilized, blocked, and incubated with anti-HMGB1 primary and corresponding secondary antibodies. For CRT staining, fixed and blocked cells (without permeabilization) were incubated with anti-CRT primary and secondary antibodies. All images were taken with CLSM. Culture supernatants were collected and assayed for ATP using a commercial kit.

### 2.15 *In vitro* dendritic cell maturation assay

4T1 cells (drug-pretreated) and BMDCs (from 6-8-week-old male C57BL/6J mice, maintained in RPMI 1640 with 10% FBS and 20 ng/mL GM-CSF) were co-cultured in 12-well plates. Then, supernatant BMDCs were collected, stained with anti-CD11c-APC, CD80-FITC, and CD86-PE, and analyzed by flow cytometry for maturation markers.

### 2.16 *In vivo* biodistribution

Tumor-bearing mice were randomly divided into three groups and received intravenous injections of free DiR, DiR-OC, or DiR-OC@cRGD, respectively. The mouse model was established by injecting 4 × 10^6^ 4T1 cells (in 100 μL PBS) into the mammary fat pad of female BALB/c mice; the aforementioned drug administration was performed once the tumor volume reached 100 mm^3^. Imaging was performed using the IVIS Spectrum system 2 h and 24 h after injection. Mice were euthanized per ethical guidelines at study end.

### 2.17 *In vivo* antitumor efficacy

As described in Section 2.16, an in situ 4T1 breast tumor model was established. When tumor volumes reached approximately 100 mm^3^, tumor-bearing mice were randomly assigned to seven groups: G1, Control; G2, Orlistat + Ce6; G3, OC; G4, OC@cRGD; G5, Orlistat + Ce6 +L; G6, OC +L; and G7, OC@cRGD +L, where L denotes laser irradiation. For groups G2–G7, the corresponding formulations were administered via tail vein injection at a Ce6-equivalent dose of 3 mg/kg and an Orlistat dose of 3 mg/kg once every 3 days. For the phototherapy groups (G5–G7), laser irradiation was applied to the tumor site on the day after injection (200 mW/cm^2^, 5 min). Tumor volume and body weight were monitored every 2 days throughout the treatment period. Tumor volumes were measured using a digital caliper and calculated as V = (L × W^2^)/2, where L and W are the longest and shortest tumor diameters, respectively. At the end of the experiment, mice were humanely euthanized in accordance with the approved ethical protocol, and tumors were excised, photographed, and weighed to further evaluate therapeutic efficacy.

### 2.18 *In vivo* antitumor immune effect evaluation

The orthotopic 4T1 mammary tumor model and dosing protocol followed the procedures outlined above. At the end of treatment, animals were sacrificed, and both tumor tissues and tumor-draining lymph nodes were collected and dissociated into single-cell suspensions for immunophenotypic analysis. To assess tumor-associated macrophages, cells isolated from tumors were stained with the viability dye eFluor 506, along with fluorophore-conjugated markers targeting CD45, F4/80, CD11b, and CD86. To examine dendritic cell maturation, cell suspensions obtained from tumors as well as draining lymph nodes were incubated with eFluor 506 and antibodies recognizing CD16/32, CD45, CD11c, IA/IE, CD80, and CD86, each coupled to the indicated fluorescent tags. For T-lymphocyte profiling, cells isolated from the same tissues were stained with eFluor 506 and fluorescently labeled probes targeting CD16/32, CD45, CD3, CD4, and CD8. The resulting samples were subjected to flow cytometry, and the acquired data were analyzed using FlowJo software (version 10.9.0) to characterize immune-cell infiltration and activation status in tumors and their draining lymph nodes.

### 2.19 mRNA sequencing and analysis

Tumor samples used for transcriptomic analysis were collected from mice subjected to the same orthotopic model construction and treatment schedule described above. After sacrifice, tumor tissues were excised for mRNA sequencing. Total RNA was isolated with TRIzol reagent per the manufacturer's instructions. Following RNA quality assessment, library construction and high-throughput sequencing were performed by Sanshu Biotechnology Co., Ltd. (https://www.sanshubio.com/). Downstream bioinformatic analysis was conducted on the Majorbio Cloud Platform (https://www.bioinformatics.com.cn/).

### 2.20 Statistical analysis

Data are expressed as mean ± standard deviation (SD). GraphPad Prism software (version 10.1.2, GraphPad Software, USA) served as the tool for statistical processing. For comparisons involving three or more groups, one-way or two-way analysis of variance (ANOVA) was used, supplemented by Tukey's multiple comparison test as appropriate; differences between two groups were assessed using the unpaired Student's t-test. The figure legends specify the n value, which represents independent biological replicates or the number of animals. A p-value of less than 0.05 is used as the threshold for statistical significance.

## 3 Results and discussion

### 3.1 Preparation and characteristic analysis of OC and OC@cRGD NPs

The initial drug feeding ratio was first examined to determine its influence on nanoparticle formation. As the Ce6 mass fraction increased, the hydrodynamic diameter gradually increased, whereas changing the proportion of orlistat caused little variation in particle size (Fig. 1A). To assess the intrinsic dark cytotoxicity of the free drug combination before nanoassembly, 4T1 cells were incubated with free mixed orlistat and Ce6 at different mass ratios. A higher Ce6 proportion led to increased dark cytotoxicity, while varying the orlistat proportion produced little effect on cell viability (Fig. 1B). Based on these physicochemical and biological results, an orlistat-to-Ce6 mass feeding ratio of 1:1 was chosen for subsequent studies.

**Fig. 1:**
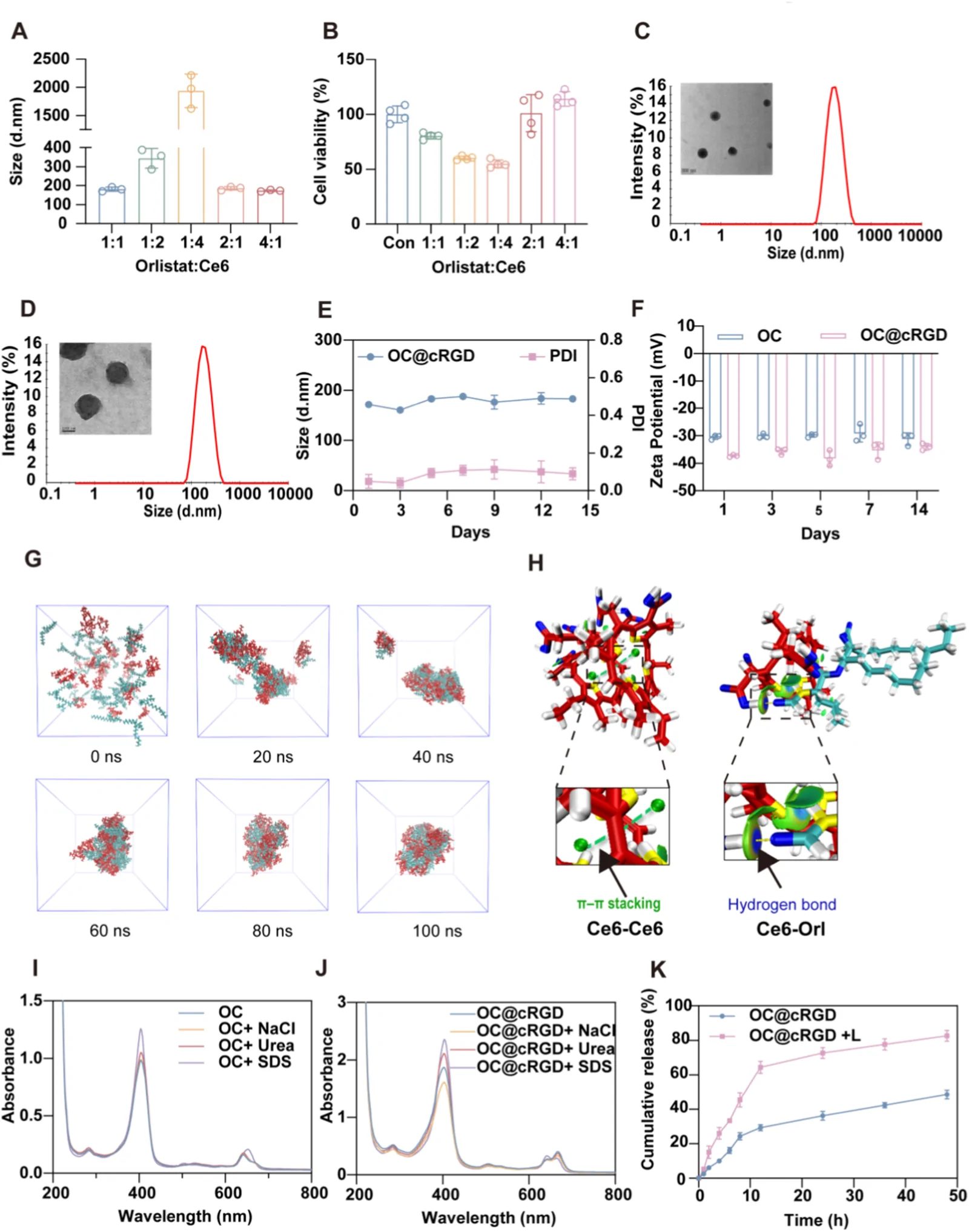
Preparation and characterization of the self-assembled nanomedicine OC@cRGD. (A) Hydrodynamic diameters of OC nanoassemblies prepared at various orlistat/Ce6 mass feeding ratios. (B) Relative viability of 4T1 cells following incubation with free mixed orlistat and Ce6 at different mass ratios. (C) OC TEM image and DLS particle size distribution plot. Scale bar = 200 nm. (D) OC@cRGD TEM image and DLS particle size distribution plot. Scale bar = 100 nm. (E) Colloidal stability of OC@cRGD over a 14-day storage period, evaluated by changes in particle diameter and polydispersity index. (F) Variations in zeta potential for OC and OC@cRGD during storage. (G) Representative molecular dynamics snapshots showing the spontaneous co-assembly process of orlistat and Ce6 within 100 ns. (H) Enlarged view of the principal intermolecular forces involved in nanoassembly, including π–π interactions and hydrogen bonding. (I, J) UV–vis spectra of OC and OC@cRGD after incubation with NaCl, urea, or SDS. (K) In vitro cumulative release behavior of Ce6 from OC@cRGD under dark conditions and after exposure to a 660 nm laser.

Using this ratio, OC nanoparticles were successfully formed through the self-assembly of orlistat and Ce6, and TEM images confirmed well-dispersed spherical nanostructures (Fig. 1C). The amount of DSPE-PEG2000-cRGD used for surface modification was then optimized by preparing nanoparticles with Ce6:Orlistat:DSPE-PEG2000-cRGD mass ratios of 2:2:0.5, 2:2:1, and 2:2:2. Particle size, PDI, and cRGD modification efficiency were compared among these formulations, with the modification efficiency quantified by the BCA protein assay using a cRGD standard curve (Fig. S2I). As shown in Table S1, the 2:2:1 formulation achieved the best overall performance, with a relatively small particle size, the lowest PDI, and a high cRGD modification efficiency (73.17 ± 2.16%), and was therefore selected for further study. DLS analysis showed that OC had an average hydrodynamic diameter of 166.3 ± 2.1 nm. After cRGD decoration, OC@cRGD maintained a similar spherical morphology (Fig. 1D), while its hydrodynamic diameter slightly increased to 171.6 ± 1.5 nm, supporting successful surface modification. In parallel, the zeta potential shifted from −30.5 ± 0.8 mV for OC to −37.3 ± 0.4 mV for OC@cRGD (Fig. 1F). Both formulations displayed good colloidal stability, with minimal changes in particle size, PDI, and zeta potential during 14 days of storage (Fig. 1E, F, and Fig. S2A). In addition, the nanoparticles remained stable for 48 h in physiologically relevant media, including 5% glucose, 10% FBS, and 0.9% NaCl (Fig. S2B and C).

To examine the non-covalent interactions responsible for nanoparticle assembly, different disrupting agents were introduced. NaCl and urea caused only minor changes, but SDS treatment led to a marked increase in particle size (Fig. S2D and E) and distinct alterations in the UV–vis absorption spectra (Fig. 1I and J). These results indicate that hydrophobic interactions are the primary driving force for nanoparticle stabilization. Molecular dynamics (MD) simulations corroborated this structural interpretation. Over a 100 ns simulation trajectory, initially dispersed orlistat and Ce6 molecules spontaneously coalesced into compact supramolecular aggregates (Fig. 1G). Structural analysis revealed robust π-π stacking interactions among the Ce6 porphyrin rings, seamlessly integrated with intermolecular hydrogen bonding between Ce6 and orlistat (Fig. 1H). Quantitative measurements found that the RMSD reached a plateau after early equilibration, while the radius of gyration and solvent accessible surface area gradually decreased (Fig. S2M–P).

Quantitative HPLC analysis demonstrated remarkable encapsulation efficiencies of 87.20 ± 4.22% for orlistat and 91.89 ± 2.27% for Ce6, corresponding to high drug loadings of 34.12 ± 1.47% and 36.27 ± 1.29%, respectively (Fig. S2K and L). Furthermore, OC@cRGD exhibited a stimuli-responsive release profile; the release kinetics of both Ce6 and orlistat were significantly accelerated under 660 nm laser irradiation compared to dark conditions (Fig. 1K and Fig. S2J). This photo-responsive behavior is likely associated with the photodynamic activation of Ce6. Upon 660 nm irradiation, excited Ce6 generates ROS, which may disturb the local supramolecular microenvironment and weaken the noncovalent interactions, including hydrophobic association, π–π stacking, and hydrogen bonding, that stabilize the co-assembled structure [42]. In addition, photoexcitation of Ce6 may alter its intermolecular packing within the nanoassembly, further loosening the assembly and promoting the release of both drugs. These results suggest that laser irradiation induces partial destabilization of the drug co-assembled nanostructure, thereby enabling on-demand payload release.

### 3.2 Cellular uptake and *in vitro* cytotoxicity

To justify the selection of cRGD as a targeting ligand, CD61 expression was examined in 4T1 tumor cells and normal mammary epithelial MCF-10A cells. CD61 expression was higher in 4T1 cells than in MCF-10A cells (Fig. S3), indicating a relatively higher integrin-related recognition background in tumor cells and supporting the use of cRGD for active targeting. Given that efficient cellular internalization is a prerequisite for targeted metabolic disruption and subsequent photodynamic therapy, we next assessed whether the elevated integrin background in 4T1 cells could be leveraged by cRGD functionalization to improve cellular uptake. After 6 h of incubation, CLSM showed only weak intracellular fluorescence in cells treated with free Ce6. In contrast, OC and OC@cRGD produced much stronger intracellular Ce6 signals, with OC@cRGD exhibiting the highest fluorescence intensity among the tested groups (Fig. 2A). Flow cytometric quantification corroborated these observations (Fig. 2B), confirming that the supramolecular nanoassembly significantly improved Ce6 delivery, while cRGD modification further enhanced active internalization via integrin-mediated endocytosis.

**Fig. 2:**
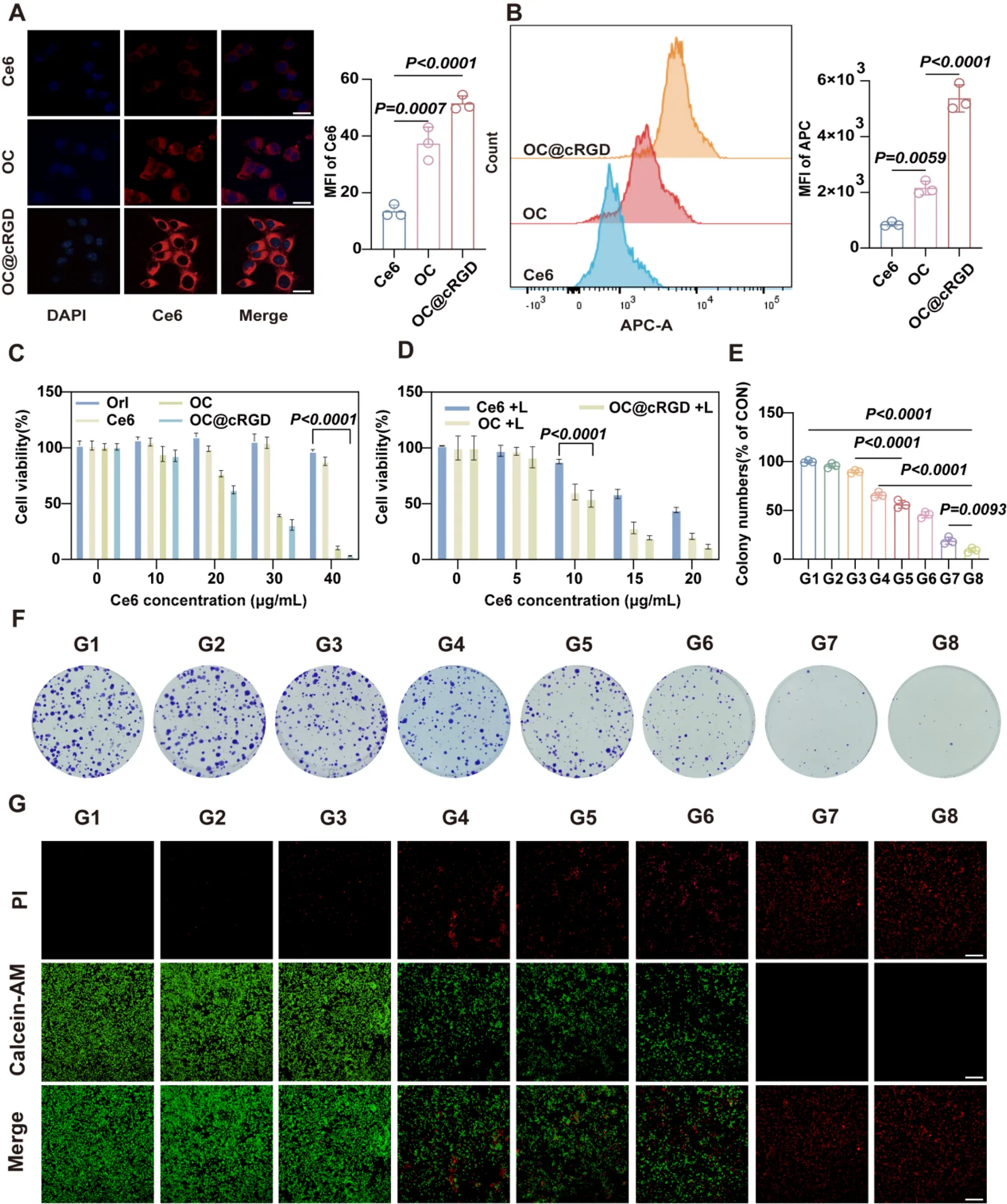
*In vitro* assessment of targeted cellular uptake and synergistic photodynamic cytotoxic effects. (A) Confocal images of 4T1 cells after 6 h of co-incubation with different formulations; intracellular Ce6-associated fluorescence was quantified. Scale bar = 10 μm. (B) Ce6 uptake in cells treated with the indicated samples was measured by flow cytometry. (C, D) Detection of 4T1 cell viability under different treatment conditions using the CCK-8 assay, without irradiation (C) or with 660 nm laser exposure (D). (E, F) Colony formation by 4T1 cells after the indicated interventions was analyzed statistically (E) and imaged representatively (F). (G) The 4T1 cells treated with different conditions were stained with Calcein-AM and PI, respectively (green: live cells; red: dead cells); the representative images show the staining of live and dead cells. Scale bar = 200 μm. The statistical analysis was accomplished using GraphPad Prism (version 10.1.2). Results are expressed as mean ± SD (n = 3). One-way ANOVA with Tukey's post hoc test was employed to determine statistical significance. (G1: Control; G2: Orlistat; G3: Ce6; G4: OC; G5: OC@cRGD; G6: Ce6 +L; G7: OC +L; G8: OC@cRGD +L). (For interpretation of the references to colour in this figure legend, the reader is referred to the Web version of this article.)

Under dark conditions, free orlistat and Ce6 caused little inhibition of cell growth, whereas both OC and OC@cRGD exhibited concentration-dependent cytotoxicity (Fig. 2C). The observed dark toxicity was mainly attributed to the intracellular delivery of orlistat, which disrupted lipid metabolism, while the co-loaded Ce6 remained largely inactive in the absence of light irradiation. Although OC@cRGD showed higher cellular uptake than OC, no statistically significant difference in antiproliferative activity was observed between the two formulations. A similar trend was also found after 660 nm laser irradiation (Fig. 2D), where OC@cRGD +L displayed the strongest overall phototoxicity among all groups, although the difference between OC@cRGD +L and OC +L did not reach statistical significance at some concentrations. These results suggest that the enhanced cellular uptake afforded by cRGD modification did not directly translate into a statistically significant increase in cytotoxicity under the present in vitro conditions. This may be attributed to several factors. First, cRGD mainly serves as a targeting ligand that promotes integrin-mediated recognition and internalization, rather than directly increasing the intrinsic cytotoxicity of the loaded drugs [43]. Second, in conventional two-dimensional cell culture systems, untargeted nanoparticles can also be internalized through nonspecific adsorption and endocytosis after incubation, thereby reducing the apparent difference between targeted and untargeted formulations [44]. Third, the relationship between nanoparticle uptake and biological effect is not necessarily linear; once the intracellular concentration of the active agents reaches an effective threshold, a moderate increase in uptake may not be sufficient to produce a statistically significant additional decrease in cell viability [45]. Nevertheless, the laser-triggered groups clearly demonstrated the therapeutic advantage of the co-delivery strategy. OC@cRGD +L consistently showed the strongest killing tendency, supporting a synergistic mechanism in which cRGD-mediated targeting facilitates intracellular Ce6 accumulation for efficient ROS generation, while orlistat-induced metabolic perturbation sensitizes the cells to oxidative stress. Colony formation assays (Fig. 2E and F) and live/dead staining (Fig. 2G) further supported this interpretation, showing that the targeted supramolecular platform combined with laser irradiation nearly abolished the long-term proliferative capacity of 4T1 cells and induced extensive cell death. Therefore, although the benefit of cRGD modification was not fully reflected as a statistically significant difference in the short-term 2D *in vitro* cytotoxicity assay, it still contributed to the overall therapeutic tendency and may become more evident *in vivo*, where active targeting can further enhance tumor accumulation, retention, and antitumor efficacy.

### 3.3 PDT and metabolic reprogramming synergistically induce ferroptosis in TNBC

To systematically explain the metabolic-redox cascade triggering ferroptosis, intracellular oxidative stress, iron accumulation, lipid peroxidation, and corresponding protein expressions were assessed. Intracellular ROS levels surged significantly upon laser irradiation, peaking in the OC@cRGD +L group (Fig. 3A–D). Because ferroptosis execution is strictly dependent on iron-catalyzed lipid peroxidation, intracellular Fe^2+^ and lipid ROS levels were subsequently quantified. Both parameters were maximally elevated in the OC@cRGD +L group (Fig. 3B, C, E). This simultaneous increase indicates that the targeted nanosystem caused more than general oxidative injury. It generated a cellular state favorable for ferroptosis, marked by severe lipid peroxidation associated with iron accumulation. TEM further showed typical mitochondrial changes linked to ferroptosis in OC@cRGD +L treated cells, including prominent shrinkage, increased membrane density, and cristae disintegration (Fig. 3F).

**Fig. 3:**
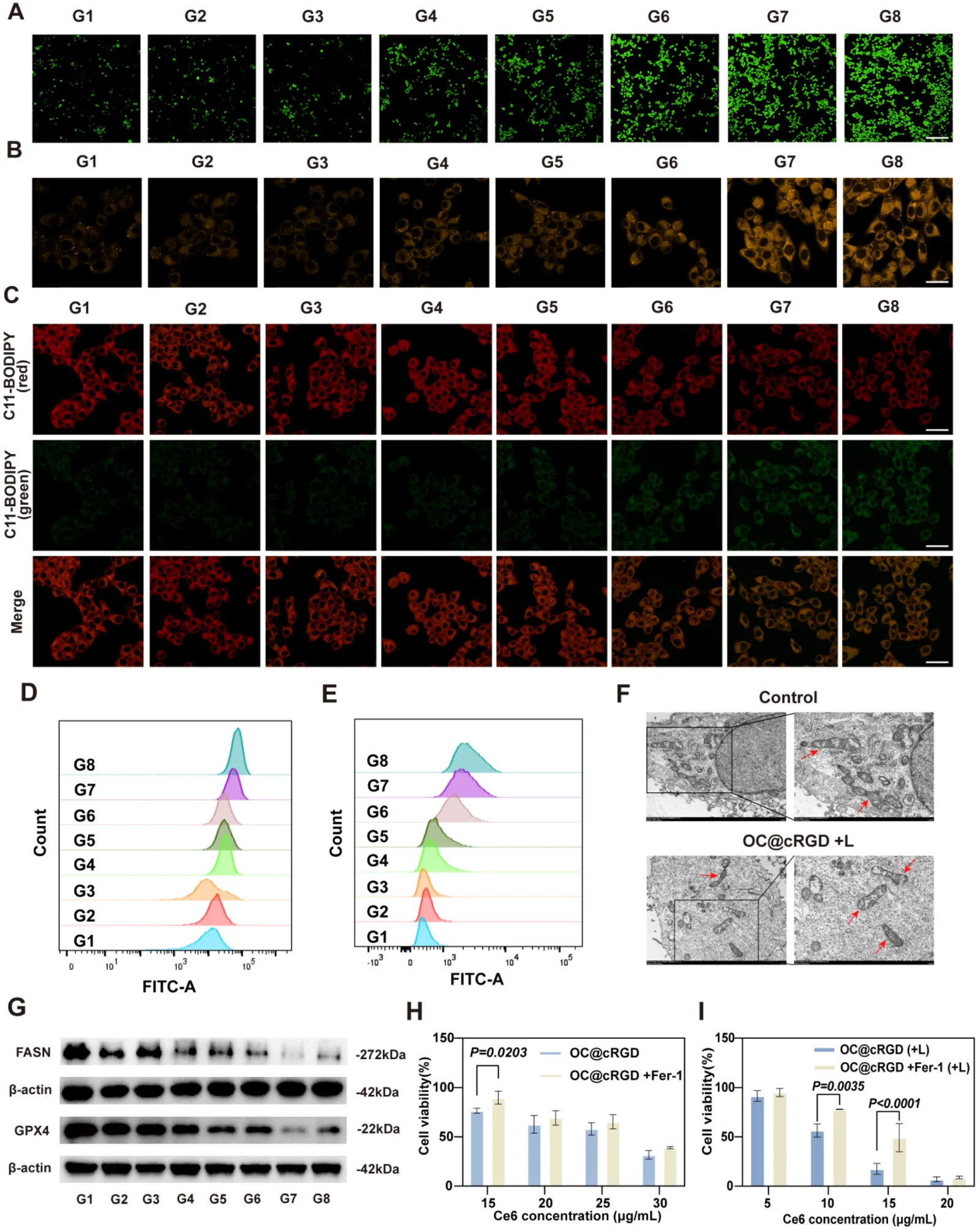
Targeted metabolic-redox nanoplatform induces ferroptosis in 4T1 cells. (A, D) Representative fluorescence images (A) and corresponding flow cytometric quantification (D) of intracellular ROS probed by DCFH-DA. Scale bar = 200 μm. (B) Intracellular labile Fe^2+^ accumulation visualized via FerroOrange staining. Scale bar = 10 μm. (C, E) C11-BODIPY probe was applied to evaluate lipid peroxidation, with representative CLSM images (C) and corresponding quantitative data (E) presented. Scale bar = 10 μm. (F) Representative TEM images delineating mitochondrial ultrastructural morphology in OC@cRGD +L-treated 4T1 cells. Red arrows highlight characteristic ferroptotic mitochondrial aberrations, including organelle shrinkage and hyperdense membranes. Scale bar = 2 μm. (G) Western blot analysis was performed to assess the expression of GPX4 and FASN in different treatment groups, using β-actin as an internal control. (H, I) Ferroptosis rescue experiments were performed using the CCK-8 assay. (H) shows the effects on cells with and without the addition of a ferroptosis inhibitor under non-irradiated conditions, while (I) shows the effects under laser irradiation conditions. The statistical analysis was accomplished using GraphPad Prism (version 10.1.2). Results are expressed as mean ± SD (n = 3). One-way ANOVA with Tukey's post hoc test was employed to determine statistical significance. (G1: Control; G2: Orlistat; G3: Ce6; G4: OC; G5: OC@cRGD; G6: Ce6 +L; G7: OC +L; G8: OC@cRGD +L). (For interpretation of the references to colour in this figure legend, the reader is referred to the Web version of this article.)

At the molecular level, laser irradiation of the nanoparticle-treated groups elicited a substantial downregulation of both GPX4 and FASN (Fig. 3G). The depletion of GPX4 dismantles the primary antioxidant barrier against phospholipid hydroperoxides [46]. Concurrently, the suppression of FASN abolishes *de novo* lipogenesis, restricting the supply of peroxidation-resistant saturated fatty acids. To test the role of ferroptosis in this process, cells were pre-treated with Fer-1. Fer-1 partially recovered cell viability in the OC@cRGD group under dark conditions and produced a stronger rescue effect after laser irradiation (Fig. 3H and I). These results show that combining FASN inhibition with PDT sensitizes TNBC cells to ferroptosis through both metabolic disruption and oxidative stress.

### 3.4 Ferroptosis sensitization amplifies PDT-induced immunogenic cell death and dendritic cell activation

While PDT is a known inducer of ICD, the resultant danger signaling is often insufficient for robust systemic immunity. This study tested the hypothesis that ferroptosis driven by metabolic sensitization could increase PDT-induced ICD. Three classical ICD markers, including HMGB1 release, CRT exposure on the cell surface, and ATP secretion, were measured quantitatively (Fig. 4A–E). Ce6 +L caused a moderate ICD response, which matched the expected effect of conventional PDT. In contrast, the OC and OC@cRGD treatments alone exhibited baseline elevations in damage-associated molecular patterns (DAMPs), indicating that lipid metabolic disruption inherently stresses tumor cells. After laser irradiation, OC@cRGD +L produced the highest HMGB1 release, CRT membrane translocation, and ATP secretion. The coordinated increase in these DAMP signals indicates that ferroptosis sensitization acts beyond a simple additional stress input and substantially increases the immunogenic character of PDT-induced tumor cell death.

**Fig. 4:**
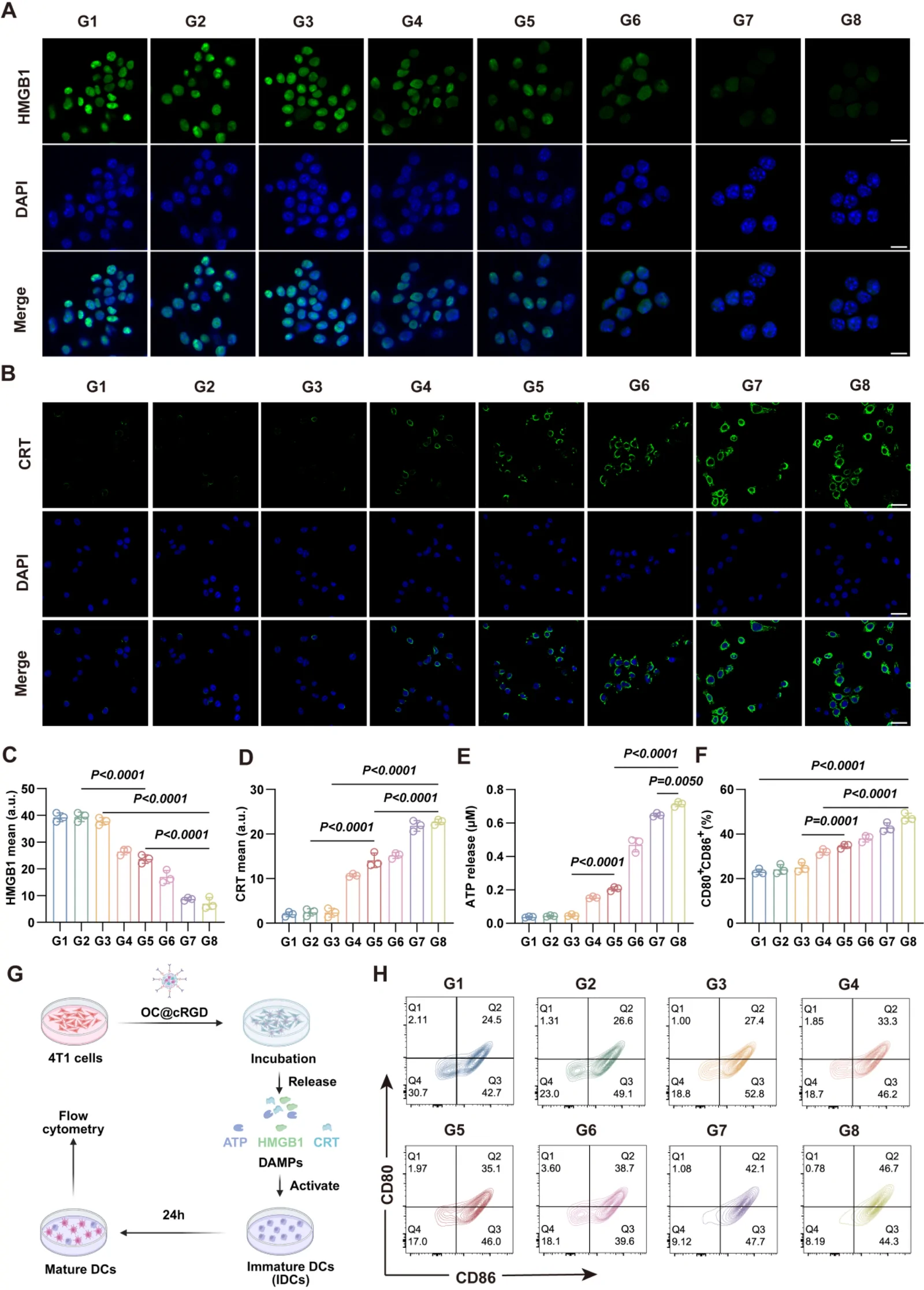
Ferroptosis sensitization amplifies PDT-induced ICD and downstream dendritic cell maturation. (A, C) HMGB1 release from treated 4T1 cells was evaluated by immunofluorescence, with representative images (A) and quantification (C) provided. Scale bar = 7.5 μm. (B, D) CRT exposure on the surface of treated 4T1 cells was examined via CLSM, and representative images (B) along with quantitative analysis (D) are shown. Scale bar = 10 μm. (E) Extracellular ATP levels released from treated 4T1 cells. (F, H) Quantitative analysis (F) and representative flow cytometry plots (H) of mature dendritic cells (CD80^+^CD86^+^) after co-culture with differently treated 4T1 cells. (G) Schematic illustration of the in vitro assay used to evaluate dendritic cell maturation. The statistical analysis was accomplished using GraphPad Prism (version 10.1.2). Results are expressed as mean ± SD (n = 3). One-way ANOVA with Tukey's post hoc test was employed to determine statistical significance. (G1: Control; G2: Orlistat; G3: Ce6; G4: OC; G5: OC@cRGD; G6: Ce6 +L; G7: OC +L; G8: OC@cRGD +L).

To verify whether this stronger DAMP profile could activate antigen-presenting cells, 4T1 cells subjected to different treatments were co-cultured with immature BMDCs. Flow cytometric analysis of CD80 and CD86 expression (Fig. 4F–H) revealed that OC@cRGD +L induced the most robust DC maturation among all experimental groups. The strong correlation between the enhanced ICD phenotype and subsequent DC maturation substantiates a cooperative mechanism: orlistat-mediated lipid perturbation lowers the ferroptotic threshold, allowing Ce6-based PDT to trigger catastrophic, highly immunogenic tumor cell destruction.

### 3.5 *In vivo* tumor-targeting ability of OC@cRGD

To evaluate biodistribution *in vivo*, mice bearing 4T1 tumors received intravenous injections of free DiR, DiR-OC, or DiR-OC@cRGD. *In vivo* fluorescence imaging showed that free DiR spread in a nonspecific manner, whereas DiR-OC accumulated moderately at the tumor site through passive targeting. DiR-OC@cRGD produced the strongest tumor fluorescence, and the signal remained clear at 24 h after injection (Fig. 5A). *Ex vivo* imaging of resected tumors and quantitative analysis of major organs further confirmed its stronger tumor-homing ability (Fig. 5B–E). Although hepatic and splenic fluorescence was observed due to inevitable reticuloendothelial system (RES) clearance, DiR-OC@cRGD maintained significantly higher tumor retention than its non-targeted counterparts, validating the efficacy of cRGD-integrin-mediated active targeting.

**Fig. 5:**
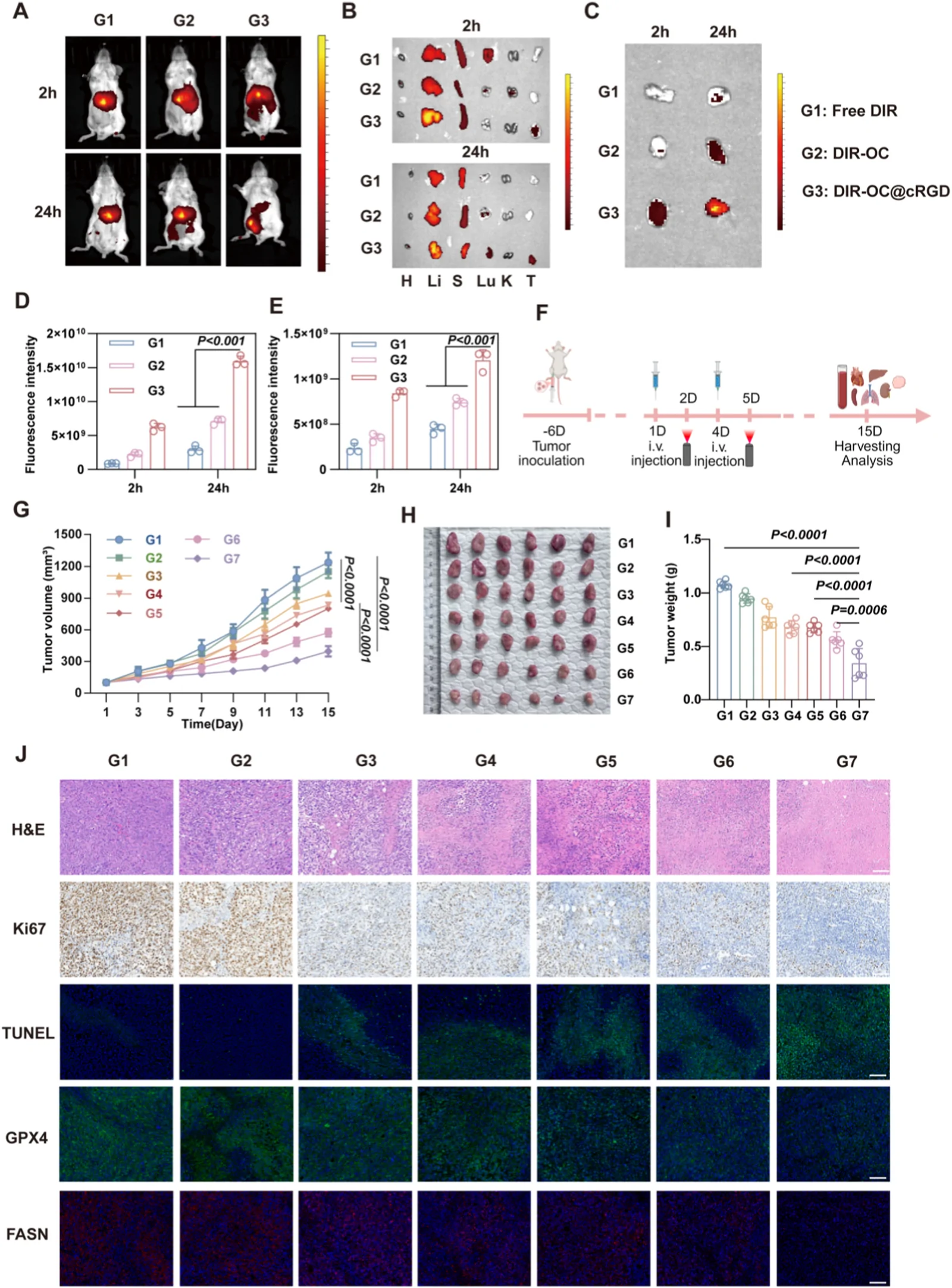
*In vivo* tumor targeting and antitumor efficacy of OC@cRGD +L in an orthotopic TNBC model. (A) 4T1 tumor-bearing mice were intravenously injected with Free DiR, DiR-OC, or DiR-OC@cRGD, and *in vivo* fluorescence images were acquired at 2 h and 24 h post-injection. (B) *Ex vivo* fluorescence images of major organs and tumors collected at 2 h and 24 h. (C) *Ex vivo* fluorescence images of excised tumors at 2 h and 24 h. (D) Quantification of *in vivo* tumor fluorescence intensity at 2 h and 24 h. (E) Quantification of *ex vivo* fluorescence intensity in excised tumors at 2 h and 24 h. (F) Schematic illustration detailing the *in vivo* treatment timeline and experimental regimen. (G) Comparative tumor growth kinetics across diverse treatment cohorts. The statistical analysis was accomplished using GraphPad Prism (version 10.1.2). Results are expressed as mean ± SD (n = 6). Two-way (repeated measures) ANOVA with Tukey's post hoc test was employed to determine statistical significance. (H) Photographic representation of excised 4T1 tumors harvested at the experimental endpoint. (I) Terminal tumor weight quantification (n = 6). (J) Histological and immunofluorescence evaluations (H&E, Ki67, TUNEL, GPX4, and FASN) of tumor tissue sections from the respective groups. Scale bar = 100 μm. The statistical analysis was accomplished using GraphPad Prism (version 10.1.2). Results are expressed as mean ± SD (n = 6). One-way ANOVA with Tukey's post hoc test was employed to determine statistical significance. (G1: Control; G2: Orlistat + Ce6; G3: OC; G4: OC@cRGD; G5: Orlistat + Ce6 +L; G6: OC +L; G7: OC@cRGD +L).

### 3.6 *In vivo* antitumor efficacy and biosafety of OC@cRGD +L

Based on the strong tumor-targeting ability of OC@cRGD, its therapeutic efficacy was further evaluated in orthotopic 4T1 tumor-bearing mice (Fig. 5F). During the observation period, tumors in the control group grew rapidly. Orlistat + Ce6, OC, and OC@cRGD exhibited only moderate antitumor activity in the absence of irradiation, indicating that drug treatment alone had limited efficacy. Upon laser irradiation, both orlistat + Ce6 +L and OC +L inhibited tumor growth more effectively than their corresponding nonirradiated groups, confirming the contribution of Ce6-based PDT. Notably, OC@cRGD +L produced the strongest antitumor effect among all groups, as reflected by the slowest tumor growth rate (Fig. 5G), consistent inhibition across individual mice (Fig. S4), and the smallest tumor size and weight at the endpoint (Fig. 5H and I). Compared with OC +L, the enhanced efficacy of OC@cRGD +L further demonstrated the advantage of cRGD-mediated tumor targeting. Nevertheless, the *in vivo* therapeutic outcome of OC@cRGD +L was manifested mainly as tumor growth delay/inhibition rather than marked tumor regression. This moderate response may be attributed to several intrinsic limitations of PDT in solid tumors [8], [12], [47]. First, ROS generation by Ce6 is highly oxygen-dependent, whereas orthotopic 4T1 tumors are characterized by a hypoxic microenvironment, which can substantially restrict PDT efficacy. Second, the penetration depth of laser irradiation in solid tumor tissue is inherently limited, reducing effective activation of the photosensitizer throughout the entire tumor mass. In addition, compared with *in vitro* conditions, the *in vivo* tumor microenvironment is much more complex, and factors such as heterogeneous drug distribution, abnormal tumor vasculature, and rapid tumor progression in the aggressive 4T1 model may further compromise therapeutic outcomes. Despite these limitations, OC@cRGD +L still achieved the strongest tumor inhibition among all treatment groups, underscoring the advantage of this targeted nanoformulation in improving the *in vivo* therapeutic efficacy.

Histological and immunofluorescence analyses of excised tumors offered further mechanistic evidence. Hematoxylin and eosin (H&E) and TUNEL staining showed severe tissue necrosis and widespread apoptosis in the OC@cRGD +L group, together with a marked reduction in Ki67 positive proliferating cells (Fig. 5J). Immunofluorescence also confirmed the simultaneous downregulation of GPX4 and FASN in tumor tissue. The loss of both proteins provides *in vivo* molecular evidence that OC@cRGD +L disrupts the antioxidant defense system and lipid metabolic support that regulate ferroptosis susceptibility, while also exerting apoptosis inducing and photodynamic effects.

The biosafety of OC@cRGD +L *in vivo* was evaluated by H&E staining of major organs, including the heart, liver, spleen, lung, and kidney, as well as by hematological analysis. Histological examination found no obvious pathological abnormalities, inflammatory infiltration, or structural injury in any treatment group. In particular, organs from mice treated with OC@cRGD +L showed no detectable lesions and maintained tissue structures similar to those in the untreated control group (Fig. S6). Hematological parameters also stayed within normal physiological ranges (Fig. S5). These findings indicate that the treatment caused no apparent systemic toxicity and suggest a favorable safety profile for this self-assembled nanoplatform.

### 3.7 Evaluation of systemic immune activation *in vivo*

Based on the significant *in vitro* induction of ICD and DC maturation, flow cytometry was used to examine whether OC@cRGD +L elicits a systemic antitumor immune response *in vivo*. Tumor tissues and tumor-draining lymph nodes (TDLNs) were analyzed. Fig. S7 presents the gating strategy for each immune subset, with DC gating in panel A, T cell gating in panel B, and macrophage gating in panel C. DC maturation forms a central link between innate antigen sensing and adaptive T cell priming. After OC@cRGD +L treatment, mature DCs marked as CD86^+^CD80^+^ increased to about 54.9% in the tumor microenvironment (Fig. 6A and B). TDLNs act as major sites of antigen presentation. In line with this role, mature DCs also rose to 49.7% in TDLNs, showing that OC@cRGD +L extended immune activation beyond the local tumor site (Fig. 6C and D). This efficient antigen presentation led to strong adaptive T cell activation. OC@cRGD +L recruited a large population of cytotoxic T lymphocytes, and CD8^+^ T cells accounted for 45.2% of the tumor infiltrating T cell population (Fig. 6E and F). Flow cytometry also recorded a clear expansion of CD8^+^ T cells in TDLNs (Fig. 6G and H), confirming that DC dependent priming generated a systemic CTL response. Activated CD8^+^ T cells then entered the tumor bed. Together with residual DAMPs released during ferroptotic ICD, these cells reshaped innate immunity within the tumor microenvironment. OC@cRGD +L significantly increased proinflammatory M1 like macrophage polarization (CD11b^+^CD86^+^) to 53.4% (Fig. 6I and J). This result indicates that treatment reversed the naturally immunosuppressive state of the TNBC microenvironment. Taken together, these *in vivo* immune profiles show that targeted ferroptotic photoimmunotherapy drives a coordinated antitumor immune process, including DC maturation, CTL priming, and macrophage repolarization. In this way, local metabolic and oxidative tumor destruction develops into sustained systemic T cell mediated immune surveillance.

**Fig. 6:**
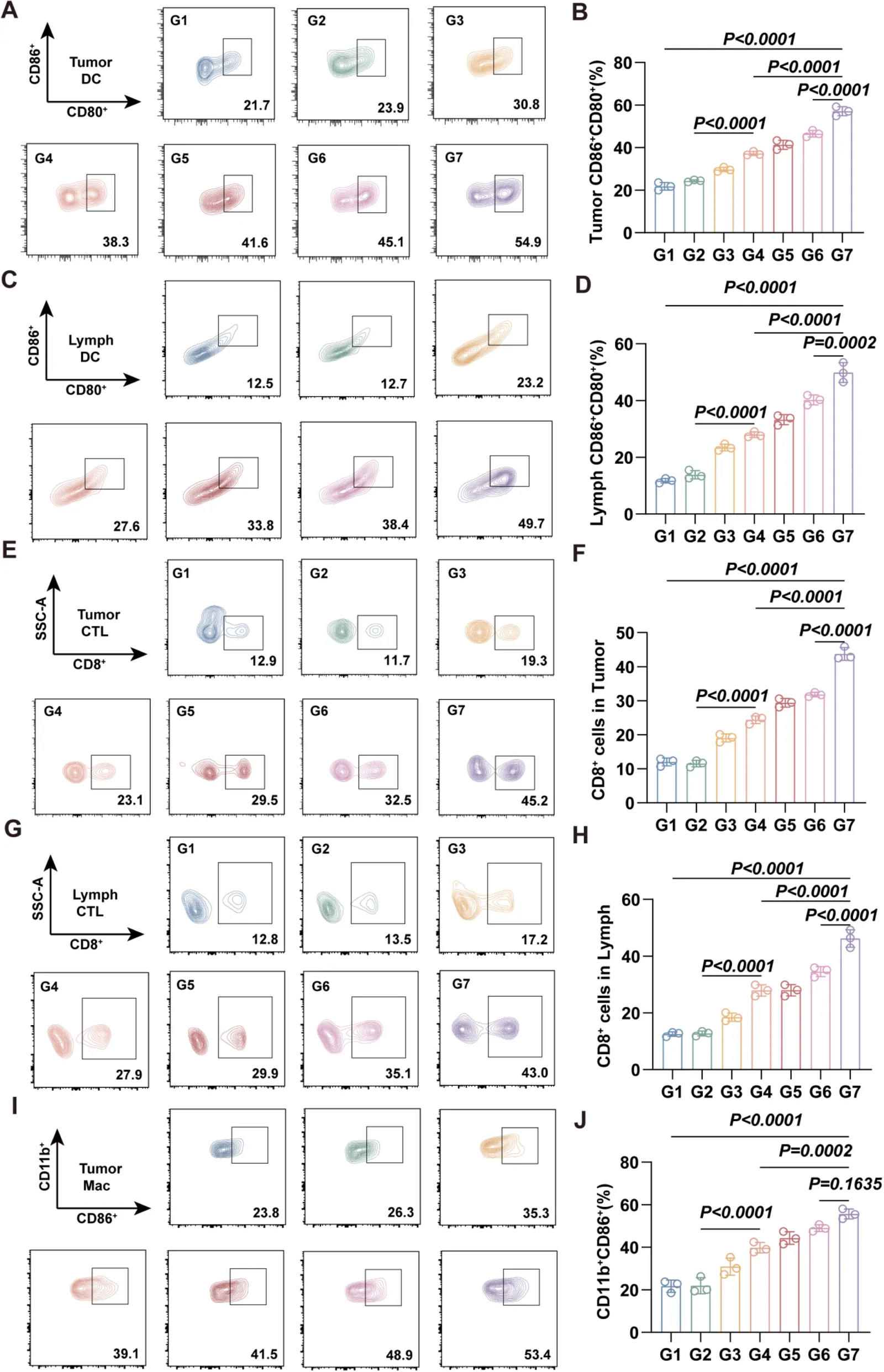
*In vivo* systemic immune remodeling and adaptive T-cell activation. (A, B) The characterization of mature dendritic cells (CD80^+^CD86^+^) in the tumor microenvironment using a representative flow cytometry scatter plot (A), followed by quantitative statistical analysis (B). (C, D) Flow cytometric characterization of mature DCs in TDLNs (C) and the results of statistical quantification (D). (E, F) Representative plots (E) and corresponding analysis (F) of cytotoxic T lymphocyte infiltration (CD8^+^) within tumors. (G, H) Flow cytometric evaluation (G) and quantification (H) of CTL expansion within the TDLNs following intervention. (I, J) Flow cytometric assessment (I) and statistical analysis (J) of M1-like macrophage (CD11b^+^CD86^+^) polarization within the tumor tissues. The statistical analysis was accomplished using GraphPad Prism (version 10.1.2). Results are expressed as mean ± SD (n = 3). One-way ANOVA with Tukey's post hoc test was employed to determine statistical significance. (G1: Control; G2: Orlistat + Ce6; G3: OC; G4: OC@cRGD; G5: Orlistat + Ce6 +L; G6: OC +L; G7: OC@cRGD +L).

### 3.8 Transcriptomic profiling reveals coordinated reprogramming of immune activation and ferroptosis-associated pathways

To obtain a broader mechanistic view at the whole-tumor level, transcriptome sequencing was performed on 4T1 tumor samples. Compared with the control group, OC@cRGD +L treated tumors showed clear shifts in gene expression, indicating extensive transcriptional remodeling. Differentially expressed genes (DEGs) were clustered in immune-related pathways, with Cd8a, Cd28, and Stat4 identified, and were also found in programs related to oxidative stress and ferroptosis, involving Alox15, Nos2, Hmox1, and Smox (Fig. 7A). A chord plot connected these differentially expressed genes with several biological pathways, showing a regulatory network that links inflammatory responses, reactive oxygen species signaling, and disturbed lipid metabolism (Fig. 7B). Kyoto Encyclopedia of Genes and Genomes (KEGG) and Gene Ontology (GO) enrichment analyses were performed, revealing strong enrichment in T cell activation, cytokine to cytokine receptor interaction, ferroptosis, and lipid localization pathways (Fig. 7C and D). Gene Set Enrichment Analysis (GSEA) further demonstrated significant enrichment of neutrophil extracellular trap formation, the NF-kappa B signaling pathway, and immune response-activating cell surface receptor signaling (Fig. 7E–G). Overall, these transcriptomic results indicate that OC@cRGD +L triggers ferroptosis-related lipid oxidative stress within the tumor while reshaping both innate and adaptive immune programs in the tumor microenvironment.

**Fig. 7:**
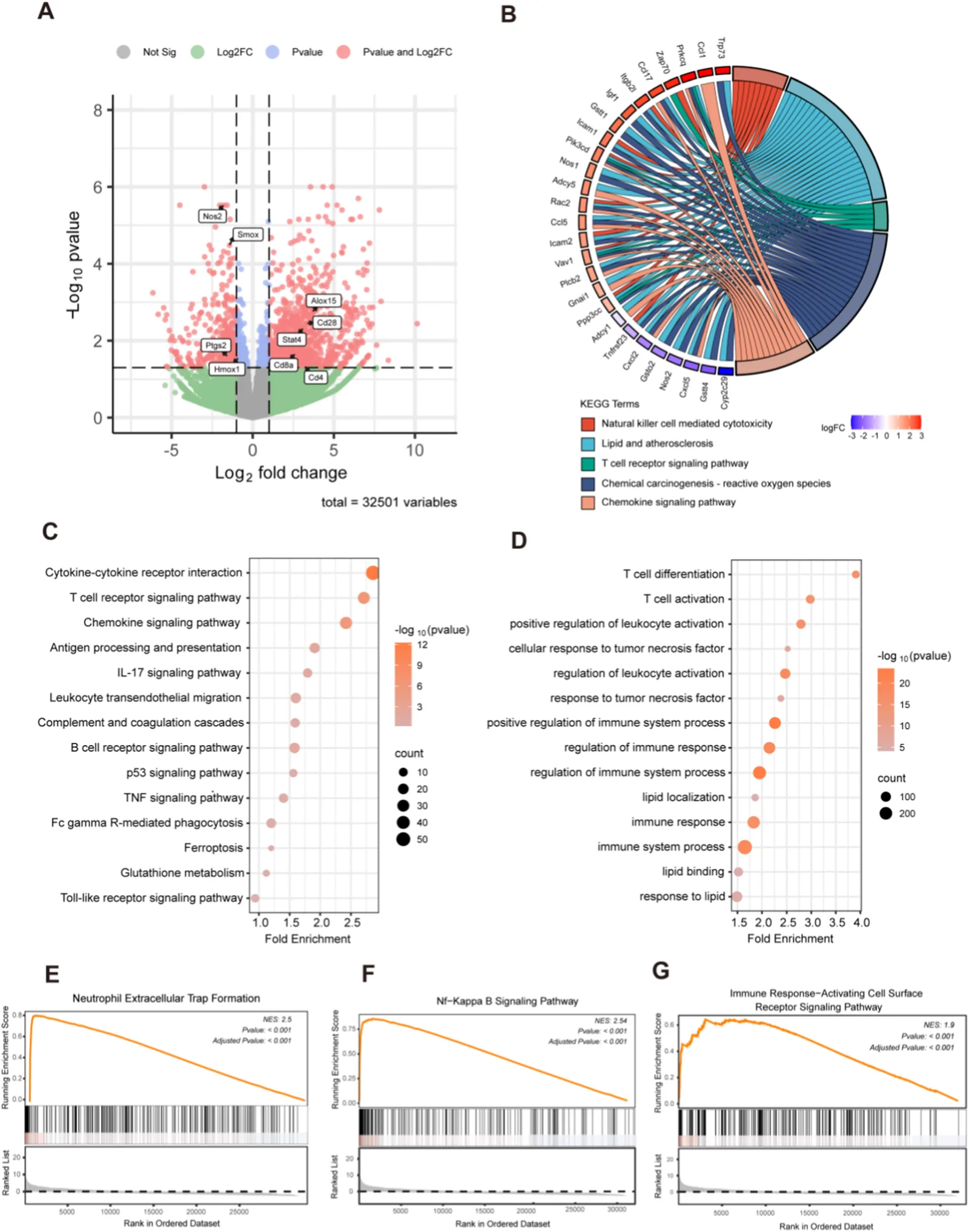
Transcriptomic characterization of 4T1 tumors after targeted photoimmunotherapy. (A) Volcano plot delineating DEGs in the OC@cRGD +L treatment cohort relative to the control. Representative genes governing immune activation, oxidative stress, and ferroptotic responses are explicitly highlighted. (B) Chord diagram illustrating the multidimensional functional interplay between specific DEGs and five critical biological pathways. The outer heatmap rim denotes log2 fold-change values. (C) The DEGs were subjected to KEGG pathway enrichment analysis. (D) The GO enrichment analysis revealed pathways associated with immune and lipid regulation. (E–G) GSEA plots showing significant enrichment of neutrophil extracellular trap formation (E), NF-kappa B signaling (F), and immune response-activating cell surface receptor signaling (G) in the OC@cRGD +L-treated group.

## 4 Conclusion

In summary, this study developed a targeted supramolecular self-assembled nanoplatform, OC@cRGD, that combines fatty acid metabolic intervention with photodynamic immunotherapy for TNBC treatment. Complementary noncovalent interactions guide the spontaneous coassembly of orlistat and Ce6, avoiding the use of inert excipients. This design increases the active therapeutic payload and maintains strong structural stability and biocompatibility. The therapeutic effect of OC@cRGD arises from a coupled metabolic and redox cascade. Orlistat targets FASN and disrupts lipid metabolic homeostasis, thereby removing the saturated fatty acid based protection that helps TNBC cells resist lipid peroxidation. At the same time, spatially and temporally controlled activation of Ce6 produces a strong oxidative burst. This process drives extensive iron dependent lipid peroxidation, mitochondrial dysfunction, and rapid GPX4 downregulation, leading to irreversible ferroptosis. Importantly, this metabolically sensitized ferroptotic damage showed strong immunogenicity. *In vivo*, OC@cRGD +L slowed tumor growth while maintaining acceptable systemic biosafety. The treatment also reshaped the immunosuppressive tumor microenvironment, as shown by dendritic cell maturation, M1 like macrophage polarization, and marked infiltration of cytotoxic CD8^+^ T cells. Overall, these findings emphasize the translational value of targeting lipid metabolism to lower the ferroptosis threshold and overcome the antioxidant barriers that restrict conventional PDT. OC@cRGD offers an efficient metabolism guided supramolecular strategy for developing next generation self-assembled nanotherapeutics, linking local tumor ablation with sustained systemic T cell mediated immune surveillance.

## CRediT authorship contribution statement

**Fei Li:** Writing – original draft, Visualization, Methodology, Investigation, Data curation, Conceptualization. **Junyong Wu:** Methodology, Investigation, Data curation. **Xinyan Hao:** Methodology, Investigation, Data curation. **Wenjie Xu:** Methodology, Investigation, Data curation. **Jiemin Wang:** Methodology, Investigation, Data curation. **Jiwen Zhang:** Methodology, Investigation. **Xingyu Wei:** Methodology, Investigation. **Niwen Feng:** Writing – review & editing, Investigation. **Fangyuan Liu:** Writing – review & editing, Investigation. **Dehua Liao:** Writing – review & editing, Validation, Supervision, Resources, Project administration, Funding acquisition.

## Ethics approval and consent to participate

All animal experiments were conducted under ethical approval No. 20260892 from the Institutional Animal Care and Use Committee of the Second Xiangya Hospital, Central South University, China. All procedures were performed in accordance with the institutional guidelines and relevant regulations for the care and use of laboratory animals. At the end of the experiments, the animals were euthanized by cervical dislocation.

## Declaration of competing interests

The authors declare that they have no known competing financial interests or personal relationships that could have appeared to influence the work reported in this paper.

## Data Availability

Data will be made available on request.
