# Supplementary material for: Supramolecular self-assembled nanomedicine targeting fatty acid metabolism to potentiate ferroptosis-enhanced photoimmunotherapy in triple-negative breast cancer
Source: Mater Today Bio. 2026 Oct 12;41:103748. doi: 10.1016/j.mtbio.2026.103748 (PMC13697287; doi:10.1016/j.mtbio.2026.103748)
Supplement: Supplementary file 1 [file mmc1.docx]

Supplementary Materials for

**Supramolecular Self-Assembled Nanomedicine Targeting Fatty Acid Metabolism to Potentiate Ferroptosis-Enhanced Photoimmunotherapy in Triple-Negative Breast Cancer**

Fei Li^a, b^, Junyong Wu^c^, Xinyan Hao^c^, Wenjie Xu^c^, Jiemin Wang^a, d^**,** Jiwen Zhang^a, b^, Xingyu Wei^a, b^, Niwen Feng^a, b^, Fangyuan Liu^a, b^, Dehua Liao^a*^

^a^ Department of Pharmacy, Hunan Cancer Hospital, the Affiliated Cancer Hospital of Xiangya School of Medicine, Central South University, Changsha, 410013, China.

^b^ School of Pharmacy, University of South China, Hengyang, 421099, China.

^c^ Department of Pharmacy, The Second Xiangya Hospital, Central South University, Changsha, 410011, China.

^d^ Comprehensive Health Research Centre (CHRC), NOVA Medical School, Faculdade de Ciências Médicas, NMS|FCM, Universidade NOVA de Lisboa, Lisboa, Portugal.

*Correspondence Author: Dehua Liao, [liaodehua1125@126.com](mailto:liaodehua1125@126.com).

**This file includes:** Table S1 and Figures S1 to S7.

**Supplementary Table**

**Table S1.** Physicochemical properties and cRGD modification efficiency of nanoparticles prepared with different mass ratios of Ce6, Orlistat, and DSPE-PEG2000-cRGD (n=3).

| Mass ratio of Ce6:Orlistat:DSPE-PEG2000-cRGD | Size (nm) | PDI | cRGD modification efficiency (%) |
| --- | --- | --- | --- |
| 2:2:2 | 204.33 ± 4.27 | 0.199 ± 0.027 | 76.5 ± 1.27 |
| 2:2:1 | 171.6 ± 1.5 | 0.07 ± 0.058 | 73.17 ± 2.16 |
| 2:2:0.5 | 161.77 ± 5.65 | 0.093 ± 0.055 | 56.3 ± 1.73 |

**Supplementary Figures**

**Figure S1.** Transcriptomic correlation analysis between FASN and ferroptosis-related genes in TNBC patients (GSE58812 cohort). (A) Comparative expression levels of GPX4 in FASN-high and FASN-low subgroups. (B) Spearman correlation analysis evaluating the relationship between FASN and GPX4 expression. (C) Comparative expression levels of ACSL4 in FASN-high and FASN-low subgroups. (D) Spearman correlation analysis evaluating the relationship between FASN and ACSL4 expression.

**Figure S2.** Comprehensive physicochemical characterization and molecular dynamics simulations of the nanoassemblies. (A) Long-term colloidal stability of OC monitored via hydrodynamic diameter and PDI over 14 days. (B, C) Colloidal stability of OC (B) and OC@cRGD (C) assessed in diverse physiological media (0.9% NaCl, 5% glucose, and 10% FBS). (D, E) Hydrodynamic size variations of OC (D) and OC@cRGD (E) following incubation with structurally disruptive agents (NaCl, urea, and SDS). (F) UV–vis absorption spectra of free orlistat, free Ce6, their physical mixture (O+C), OC, and OC@cRGD. (G, H) HPLC standard calibration curves for orlistat (G) and Ce6 (H). (I) Calibration curve of cRGD determined by the BCA assay. (J) Cumulative in vitro release profile of orlistat from OC@cRGD under dark conditions or 660 nm laser irradiation. (K, L) Encapsulation efficiency (EE) and drug loading capacity (DL) of orlistat and Ce6 within the self-assembled nanoparticles. (M–P) Root-mean-square deviation (RMSD) (M), radius of gyration (Rg) (N), and solvent-accessible surface area (SASA) (O) plotted as a function of time during the 100 ns molecular dynamics simulation. (P) Temporal evolution of the number of intermolecular hydrogen bonds during the simulation.

**Figure S3.** Western blot analysis of CD61 expression in MCF-10A and 4T1 cells.

**Figure S4**. Individual tumor growth kinetics. Spider plots illustrating the tumor growth trajectories of individual 4T1 orthotopic tumor-bearing mice across the seven designated treatment cohorts over the 15-day observation period. (G1: PBS; G2: Orlistat + Ce6; G3: OC; G4: OC@cRGD; G5: Orlistat + Ce6 +L; G6: OC +L; G7: OC@cRGD +L).

**Figure S5.** *In vivo* systemic biosafety evaluation. Hematological and serum biochemical parameters of 4T1 tumor-bearing mice following the completion of the respective treatment regimens. Quantitative analysis of (A) red blood cell (RBC) count, (B) platelet (PLT) count, (C) serum creatinine (CREA), (D) blood urea nitrogen (BUN), (E) alanine aminotransferase (ALT), and (F) aspartate aminotransferase (AST). The statistical analysis was accomplished using GraphPad Prism (version 10.1.2). Results are expressed as mean ± SD (n = 4). One-way ANOVA with Tukey’s post hoc test was employed to determine statistical significance. (G1: Control; G2: orlistat + Ce6; G3: OC; G4: OC@cRGD; G5: orlistat + Ce6 +L; G6: OC +L; G7: OC@cRGD +L).

**Figure S6.** Histological safety assessment. Representative hematoxylin and eosin (H&E) stained sections of major organs (heart, liver, spleen, lung, and kidney) harvested from 4T1 tumor-bearing mice across all treatment cohorts. No overt pathological abnormalities, inflammatory lesions, or structural damages were observed, indicating excellent systemic biosafety. Scale bar = 100 μm. (G1: Control; G2: orlistat + Ce6; G3: OC; G4: OC@cRGD; G5: orlistat + Ce6 +L; G6: OC +L; G7: OC@cRGD +L).

**Figure S7.** Flow cytometry gating strategies for immune profiling. (A) Dendritic cell (DC) gating: mature DCs (CD86⁺CD80⁺). (B) T cell gating: CD8⁺ cytotoxic T lymphocytes (CTLs). (C) Macrophage gating: M1-like macrophages (CD11b⁺CD86⁺).
